# Rise and Growth of Vaccination Law.—IV

**Published:** 1904-01-30

**Authors:** J. E. R. Stephens

**Affiliations:** author of "Digest of Public Health Cases," etc., etc.


### RISE AND GROWTH OF VACCINATION LAW.—IV.

By J. E. R. STEPHENS, Barrister-at-law, author of "Digest of Public Health Cases," etc., etc.

(*Continued from page* 107).

On May 29th, 1889, a Royal Commission was appointed to consider the whole matter. The original commissioners were Lord Herschell, Sir James Paget, Sir C. Dalrymple, Sir Guyer Hunter, Sir E. H. Galsworthy, Sir William Savory, Drs. Bristowe and Collins, and Messrs. C. Bradlaugh, J. S. Dugdale, Q.C., Michael Foster, J. Hutchinson, J. A. Picton, S. Whitbread, and F. Meadows White, Q.C. At the death of Mr. Bradlaugh Mr J. A. Bright was added to the commissioners.

The following were the terms of the reference:—To inquire and report as to (1) The effect of vaccination in reducing the prevalence of, and mortality from, small-pox. (2) What means, other than vaccination, can be used for diminishing the prevalence of small-pox, and how far such means could be relied on in place of vaccination. (3) The objections made to vaccination on the ground of injurious effects alleged to result therefrom, and the nature and extent of any injurious effects which do, in fact, so result. (4) Whether any, and, if so, what means should be adopted for preventing or lessening the ill effects, if any, resulting from vaccination; and whether, and, if so, by what means, vaccination with animal vaccine should be further facilitated as a part of public vaccination. (5) Whether any alterations should be made in the arrangements and proceedings for securing the performance of vaccination, and, in particular, in the provisions of the Vaccination Acts with respect to prosecutions for non-compliance with the law.

The inquiry of the commissioners was a very prolonged one. They held 136 meetings for the examination of witnesses, and 187 witnesses were examined. In addition, they caused inquiry to be made by competent persons in a large number of cases of alleged injury from vaccination, and they also caused to be made a series of complete and systematic investigations into the circumstances of local epidemics of small-pox in the Dewsbury Union in 1891–92 in London, in Warrington, and in Leicester in 1892–93; and in Gloucester in 1895–96. They also instituted inquiries into the extent and character of various small-pox outbreaks in several other localities.

The results of their inquiries are contained in several large Blue Books, while the conclusions and the reasoning upon which these were founded are contained in the Final Report, which was made in August, 1896. But the report was not unanimous. Two of the members of the commission—Dr. Collins and Mr. Picton—presented a separate report. Two others—Mr. Whitbread and Mr. J. A. Bright—signed the majority report, but at the same time objected to any form of compulsory vaccination; whilst two—Sir W. G. Hunter and Mr. J. Hutchinson—objected to the relaxation of the law to which the majority assented, and they also expressed an opinion in favour of compulsory revaccination at the age of twelve.

### EFFECT OF VACCINATION.

The first question which the Commissioners were required to answer was as to the effect of vaccination in reducing the prevalence of, and mortality from, small-pox. The answer of the majority was as follows:—

1. That it diminishes the liability to be attacked by the disease.

2. That it modifies the character of the disease, and renders it less fatal, and of a milder or less severe type.

3. That the protection it affords against attacks of the disease is greatest during the years immediately succeeding the operation of vaccination. It is impossible to fix with precision the length of this period of highest protection. Though not in all cases the same, if a period is to be fixed, it might, we think, fairly be said to cover in general a period of nine or ten years.

4. That after the lapse of the period of highest protective potency, the efficacy of vaccination to protect against attack rapidly diminishes, but that it is considerable in the next quinquennium, and possibly never altogether ceases.

5. That its power to modify the character of the disease is also greatest in the period in which its power to protect from attack is greatest, but that its power thus to modify the disease does not diminish as rapidly as its protective influence against attacks, and its efficacy during the later periods of life to modify the disease is still very considerable.

6. That revaccination restores the protection which lapse of time has diminished, but the evidence shows that this protection again diminishes, and that, to ensure the highest degree of protection which vaccination can give, the operation should be at intervals repeated.

7. That the beneficial effects of vaccination are most experienced by those in whose case it has been most thorough. We think it may fairly be concluded that where the vaccine matter is inserted in three or four places, it is more effectual than when introduced into one or two places only—and that if the vaccination marks are of an area of half a square inch, they indicate a better state of protection than if their area be at all considerably below this.

### Other Means to Prevent Small-Pox.

The second question on which the Royal Commission were requested to report was: What means, other than vaccination, can be used for diminishing the prevalence of small-pox; and how far such means can be relied on in place of vaccination?

We have no difficulty, say the majority, in answering the question, what means other than vaccination can be used for diminishing the prevalence of small-pox? We think that a complete system of notification of the disease, accompanied by an immediate hospital isolation of the persons attacked, together with a careful supervision, or, if possible, isolation for sixteen days of those who had been in immediate contact with them, could not but be of very high value in diminishing the prevalence of small-pox. It would be necessary, however, to bear constantly in mind as two conditions of success, first, that no considerable number of small-pox patients should ever be kept together in a hospital situate in a populous neighbourhood, and secondly that the ambulance arrangement should be organised with scrupulous care. If these conditions were not fulfilled, the effect might be to neutralise or even do more than counteract the benefits otherwise flowing from a scheme of isolation.

When we turn to the other branch of the inquiry, how far such means could be relied on in the place of vaccination, we find ourselves involved in questions of a much more complicated nature. We have little or no experience to fall back upon. The experiment has never been tried. The nearest approach to a trial of it has probably been in Australia. But even in the parts of that country to which we have alluded the population has not been entirely unvaccinated, though there has been a large unvaccinated class amongst it. Moreover, in applying the experience of Australia to this country, two things must be borne in mind. In the first place small-pox has only appeared from time to time, introduced from without at one or other of the ports of the country, and the several colonies of which Australia is composed are of great territorial extent, with few large centres of population. In this country small-pox is always present in some part of it. There has not been a single year without several deaths from the disease. Large centres of population are numerous, and the intercourse between them constant. In the several colonies of Australia the number of ports is not great, the vessels which enter them are comparatively speaking not numerous, and the ports from which they arrive are many days' voyage distant; and there are careful arrangements for quarantining vessels to exclude disease. The shipping which enters English ports is of vast quantity, and passengers are brought in large numbers from the Continent of Europe not only daily, but it may almost be said hourly; the voyage, too, is but brief. The other matter to be remembered is that part of the Australian system is the compulsory removal to quarantine for twenty-one days of those who have been in the house with the patient, in addition to the transfer of the patient himself to a hospital. There can be no doubt that such a system, if completely carried out, would be of the highest efficacy. But it is obvious that in this country the practical difficulties of working such a scheme in the large towns would be really insuperable, to say nothing of the difficulty of procuring legislative sanction for it.

*(To be continued.)*

